# Photosynthetic Acclimation of Shade-Grown Soybean Seedlings to a High-Light Environment

**DOI:** 10.3390/plants12122324

**Published:** 2023-06-15

**Authors:** Yahan Su, Huan Yang, Yushan Wu, Wanzhuo Gong, Hina Gul, Yanhong Yan, Wenyu Yang

**Affiliations:** 1 College of Agronomy, Sichuan Agricultural University, Chengdu 611130, China; 2 Key Laboratory of Crop Eco-Physiology and Farming System in Southwest of China, Sichuan Engineering Research Center for Crop Strip Intercropping System, Chengdu 611130, China; 3 Crop Research Institute, Chengdu Academy of Agricultural and Forestry Sciences, Chengdu 611130, China; 4 National Center of Industrial Biotechnology, Arid Agriculture University, Rawalpindi 46300, Pakistan; 5 College of Grassland Science and Technology, Sichuan Agricultural University, Chengdu 611130, China

**Keywords:** intercropping, soybean, leaf, phenotype, acclimation

## Abstract

Soybean in relay intercropping is initially exposed to a shade environment, followed by exposure to full sunlight after the harvesting of primary crops, e.g., maize. Therefore, soybean’s ability to acclimate to this changing light environment determines its growth and yield formation. However, the changes in soybean photosynthesis under such light alternations in relay intercropping are poorly understood. This study compared the photosynthetic acclimation of two soybean varieties with contrasting shade tolerance, i.e., *Gongxuan1* (shade-tolerant) and *C103* (shade-intolerant). The two soybean genotypes were grown in a greenhouse under full sunlight (HL) and 40% full sunlight (LL) conditions. Subsequently, after the fifth compound leaf expanded, half of the LL plants were transferred to a high-sunlight environment (LL-HL). Morphological traits were measured at 0 and 10 days, while chlorophyll content, gas exchange characteristics and chlorophyll fluorescence were assayed at 0, 2, 4, 7 and 10 days after transfer to an HL environment (LL-HL). Shade-intolerant *C103* showed photoinhibition 10 days after transfer, and the net photosynthetic rate (*P_n_*) did not completely recover to that under a high light level. On the day of transfer, the shade-intolerant variety, *C103*, exhibited a decrease in net photosynthetic rate (*P_n_*), stomatal conductance (*G_s_*) and transpiration rate (*E*) in the low-light (LL) and low-light-to-high-light (LL-HL) treatments. Additionally, intercellular CO_2_ concentration (*C_i_*) increased in low light, suggesting that non-stomatal factors were the primary limitations to photosynthesis in *C103* following the transfer. In contrast, the shade-tolerant variety, *Gongxuan1*, displayed a greater increase in *P_n_* 7 days after transfer, with no difference observed between the HL and LL-HL treatments. Ten days after transfer, the shade-tolerant *Gongxuan1* exhibited 24.1%, 10.9% and 20.9% higher biomass, leaf area and stem diameter than the intolerant *C103*. These findings suggest that *Gongxuan1* possesses a higher capacity to adapt to variations in light conditions, making it a potential candidate for variety selection in intercropping systems.

## 1. Introduction

Food security is a big challenge across the world owing to the changing global climate, increasing population and decreasing cultivable land [1,2]. Under these circumstances, intercropping has been employed as a sustainable agricultural practice in many areas of the world. It exhibits temporal complementarity and high resource use efficiency by utilizing light, water and nutrition [3,4]. Soybean (*Glycine max* Merr.), the fourth most widely cultivated crop worldwide, is a key component of global food security, and is the main source of plant protein and oil [5]. In order to increase the area under cultivation and yield of soybean, it is also planted in various intercropping combinations with maize, sorghum, sunflower and fruit trees [6,7,8,9]. However, soybean is often exposed to shade conditions under these intercropping systems.

The maize–soybean relay strip intercropping system is a widely practiced system worldwide [8,10,11]. In this system, maize is usually sown at the beginning of April, while soybean is planted in the middle of June; the late-planted soybean experiences varying growth environments, i.e., the shade period when maize and soybean grow simultaneously, and the natural sunlight period after the harvesting of maize [12,13]. In the shade period, the maize canopy can reduce the availability of PAR and alter the light quality for soybean seedlings. Soybean changes its morphological and physiological traits, for example, through elongation of its stem, petiole and hypocotyl, and by reducing its branching and stem diameter, to acclimatize under shade conditions and improve light capture [9,14]. The decreased leaf area under shade conditions leads to reduced biomass, as plants invest the biomass in stem elongation rather than the leaf [10,15]. Additionally, some soybean genotypes also show shade tolerance traits, such as increased specific leaf area (SLA) and chlorophyll (Chl) content and a reduction in *P*_n_ and the Chl *a*/*b* ratio [14].

However, knowledge about the photosynthetic response of soybean under different light conditions imposed by varying growth conditions under intercropping is still limited. Soybean is grown under full sunlight during the solo-growth period of soybean in the maize–soybean relay strip intercropping system after maize harvest. This period lasts around two months and cannot be neglected as it is the main period of yield formation [12,13]. During this period, studies have reported the compensatory growth of soybean, under which the leaf area and leaf mass increase rapidly, and soybean grows into the reproductive stages [13]. Similarly, the significant increase in dry matter accumulation during the recovery growth period is also an important factor contributing to the yield advantage of maize–soybean relay strip intercropping [16]. Notably, most previous studies have focused on soybean yield formation or yield components in relay intercropping at final harvest, while the underlying physiological mechanisms of soybean recovery from shade to full sunlight are yet to be elucidated. Moreover, genetic variation needs to be investigated to study soybean acclimation. It is necessary to compare the responses among different soybean varieties when transferred from low light to high light. Therefore, the current study was conducted with the objectives of: (1) testing whether soybean seedlings grown in low light can acclimate to high light, and (2) comparing the differences in photosynthetic characteristics between soybean varieties with different shade tolerance when transferred from shade after acclimatizing to high-light conditions.

## 2. Methods

### 2.1. Experimental Design

**Figure 1:**
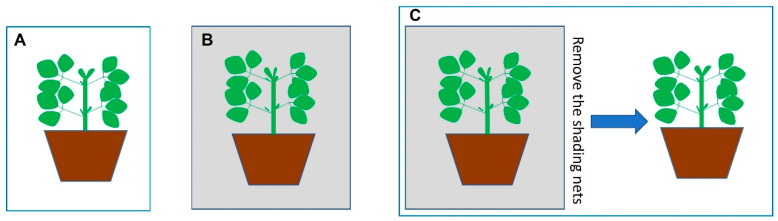
Experimental design of this study. (**A**) plant grown in high light (HL), (**B**) plant grown in low light (LL), (**C**) plant transferred from low light to high light (LL−HL). The grey background in (**B**,**C**) represents the shading nets.

The experiment was conducted in a greenhouse at the Teaching and Experimental Farm of Sichuan Agricultural University, Ya’ an (29°59′ N, 103°00′ E). Two soybean varieties, shade-tolerant *Gongxuan1* [13] and shade-intolerant *C103* [17], were selected in this study under three light treatments, with at least three replicates in each treatment. Plants were sown in polythene pots (20 cm diameter × 40 cm depth) and watered to maintain the soil at field capacity. The soil was a purple clay loam (pH 6.6), with 1.21 g kg^−1^ N, 0.61 g kg^−1^ P, 11.44 g kg^−1^ K, 62.35 mg kg^−1^ available N, 24.34 mg kg^−1^ available P, 65.72 mg kg^−1^ available K and 8.96 g kg^−1^ organic matter. The three light treatments were high light (HL), low light (LL), and low light-to-high light transfer (low light for 50 days, and then, high light for 10 days (LL-HL)) (Figure 1). Each treatment contained 20 pots per variety, and the study lasted 60 days. In HL treatments, soybean plants were allowed to grow under natural sunlight conditions; in LL treatment, soybean plants were grown under 40% full sunlight (photosynthetically active radiation was 500 μ mol m^−2^ s^−1^, measured using an LM8130 Digital Meter Illuminometer) using shading nets (Q-MAX, USA, R:FR (0.5–0.6)) above and around pots throughout the experiment. In the transfer treatment, soybean was grown under LL for 50 days, and then, 10 pots were transferred to HL to simulate light recovery after the harvest of maize in the maize–soybean relay strip intercropping system. One day before the transfer, the latest fully expanded leaf (the third compound leaf from the top) from three treatments was tagged for measurements. Different parameters were measured 0, 2, 4, 7 and 10 days after the transfer, as described below in detail.

### 2.2. Gas Exchange Characteristics

Before the destructive sampling, the latest fully expanded leaves were examined using an infrared gas analysis instrument (*LI-6400*, Li-COR Inc., Lincoln, NE, USA) from 10:00 to 14:00 h to record net photosynthetic rate (*P_n_*), stomatal conductance (*G_s_*), intercellular CO_2_ concentration (*C_i_*) and transpiration rate (*E*). At least six seedlings of each soybean variety were measured under each treatment.

### 2.3. Chlorophyll Fluorescence

After measuring gas exchange characteristics, chlorophyll fluorescence was analyzed using a PAM-2000 pulse-amplitude-modulated fluorometer (Heinz Walz GmbH, Effeltrich, Germany) following a 30 min dark adaptation period. The maximum quantum yield of PSII (*F*v/*F*m), photochemical efficiency of PSII (*Φ*PSII), electron transport rate (ETR) and non-photochemical quenching (NPQ) were determined. Photon capture efficiency was determined through open Photosystem II (PSII) reaction centers in dark-adapted foliage.

### 2.4. Chlorophyll Content

Fresh leaves of the latest fully expanded leaves were sampled and quickly brought to the laboratory; six leaf discs were punched and extracted in 80% aqueous acetone solvent to determine total Chl and Chl *a*/*b* via spectrophotometric analysis [18].

### 2.5. Growth Characteristics

Six plants per pot were sampled 0 and 10 days after transfer to HL to measure biomass, plant height, and stem diameter. The latest fully expanded leaves were scanned using a flatbed scanner (*CanoScan LiDE 200*, Canon Inc., Tokyo, Japan) and the area was measured using *ImageJ 1.45 s*. After the sampling, plants were divided into roots, leaves, petioles and stems. Total biomass was calculated after the roots, leaves, petioles and stems were oven-dried to a constant mass. SLA was calculated by dividing the measured area by its dry mass (DM), total leaf area (LA) was calculated by multiplying SLA by total leaf DM, and leaf area ratio (LAR) was calculated by dividing LA by total aboveground biomass.

### 2.6. Statistical Analysis

All data analysis was performed via analysis of variance (*ANOVA*) using IBM *SPSS 19.0* for *Windows* (*SPSS*, Armonk, NY, USA: IBM Corp). Significant differences were defined as *p* ≤ 0.05.

## 3. Results

### 3.1. Leaf Chlorophyll Content

**Figure 2:**
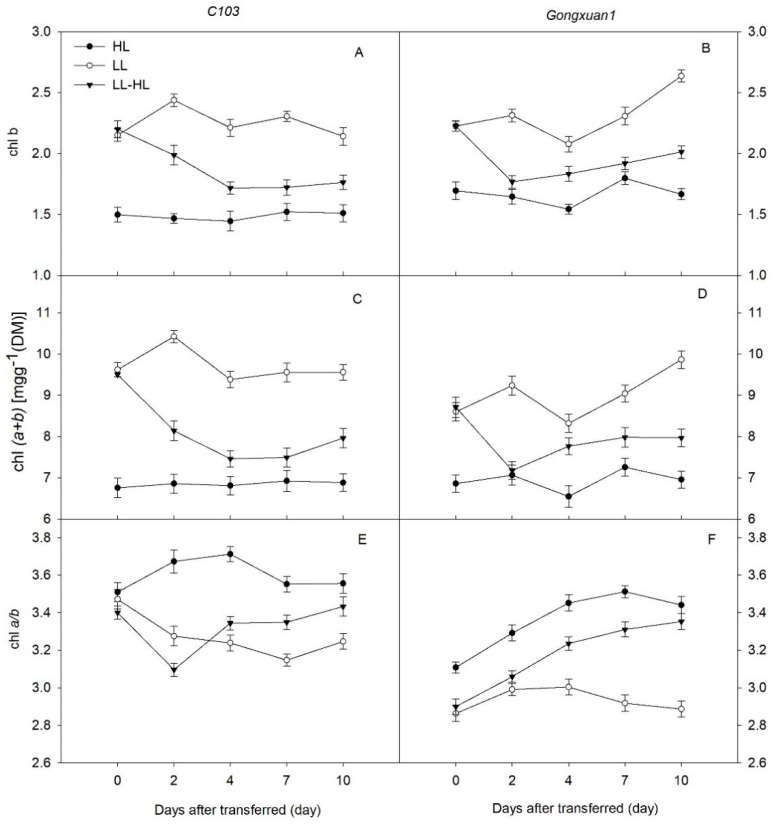
(**A**,**B**) Chl b, (**C**,**D**) total chlorophyll (Chl) (a+b), (**E**,**F**) Chl a/b of *C103* and *Gongxuan 1* under low light (LL) and high light (HL)and following transfer from low light to high light (LL−HL). Error bars show SD, *n* = 6.

Compared to HL, the total chlorophyll content (Chl (a+b)) and Chl b in LL were significantly higher, while the Chl a/b ratio was significantly lower throughout the experiment (Figure 2). Two days after the transfer to HL(LL-HL), the Chl (*a*+*b*) and Chl *b* of the two varieties decreased sharply. However, the Chl *a*/*b* ratio of the two varieties showed no significant difference between LL-HL and HL on the 10th day after transfer.

### 3.2. Gas Exchange Characteristics

**Figure 3:**
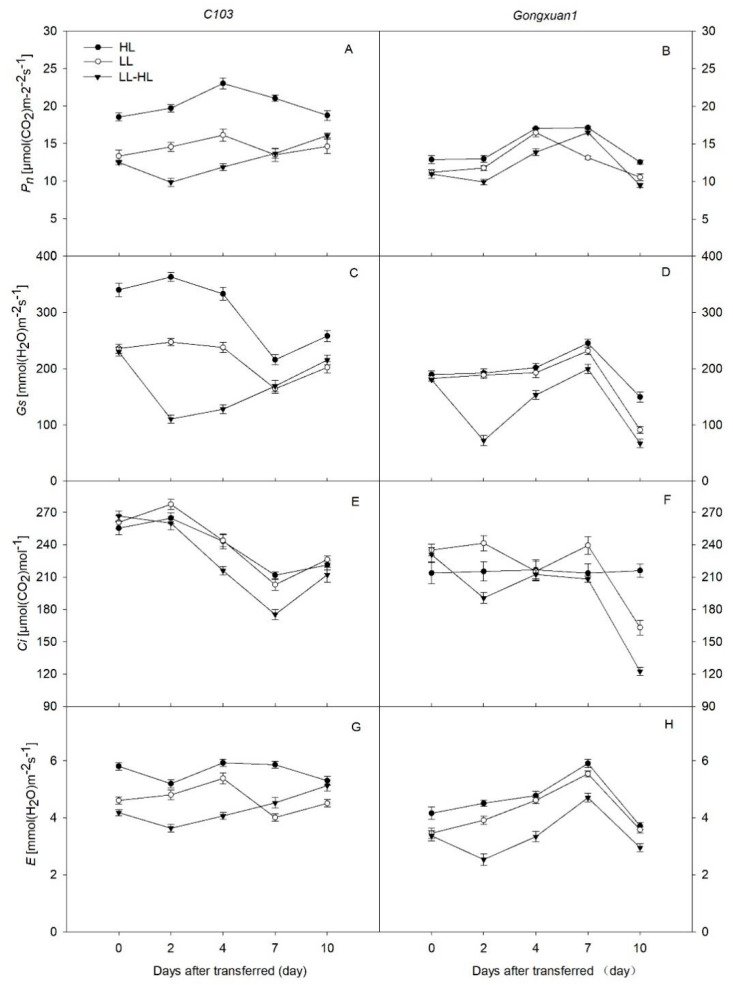
(**A**,**B**) Net photosynthetic rate (*P_n_*), (**C**,**D**) stomatal conductance (*G_s_*), (**E**,**F**) intercellular CO_2_ concentration (*C_i_*), (**G**,**H**) transpiration rate (*E*) of *C103* and *Gongxuan 1* under low light (LL) and high light (HL) and following transfer from low light to high light (LL−HL). Error bars show SD, *n* = 6.

As shown in Figure 3, two days after being transferred to high-light (HL) conditions, the photosynthetic rate (*Pn*) of *C103* seedlings growing under low-light LL-HL conditions was significantly lower (50.1%) compared to those in HL. Similar reductions were also observed in stomatal conductance (*Gs*) (69.5%) and transpiration rate (*E*) (30%) (Figure 3C,G). *Gongxuan1* seedlings exhibited a similar trend, with a decrease of 23.5% in *P_n_* and 43.8% in E under LL-HL conditions. As the recovery time increased, the *P_n_* and *G_s_* of *C103* seedlings showed some recovery, but did not reach the control level (HL). However, there was no significant difference in the *Pn* and intercellular CO2 concentration (*Ci*) in *Gongxuan1* between the LL-HL and HL conditions on the seventh day after transfer.

### 3.3. Chlorophyll Fluorescence

**Figure 4:**
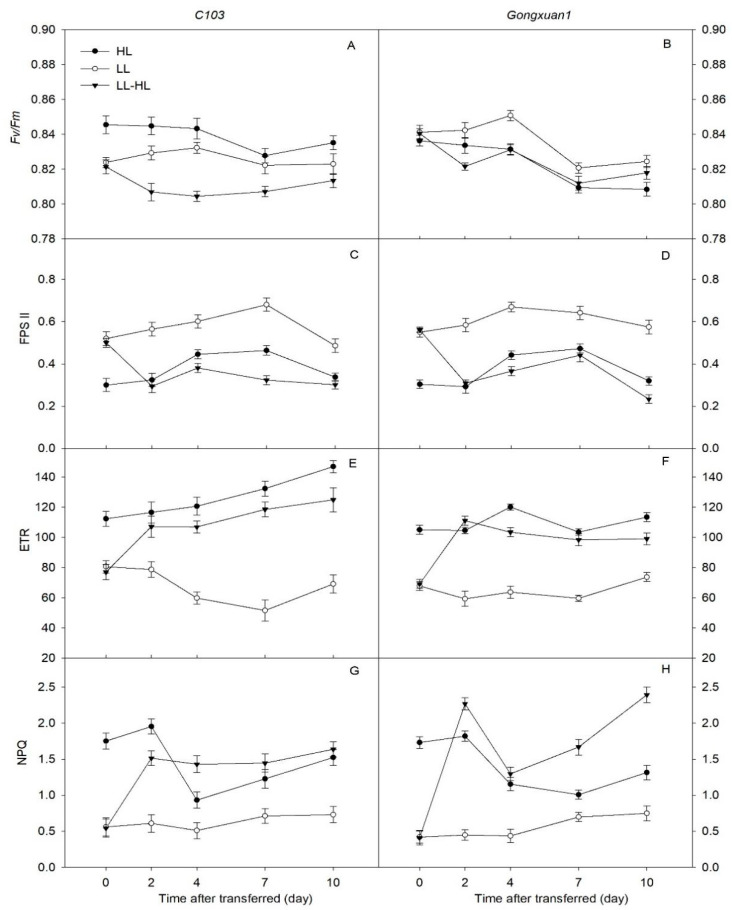
(**A**,**B**) Maximum quantum yield of PSII (Fv/Fm), (**C**,**D**) photochemical efficiency of PSII (ΦPSII), (**E**,**F**) electron transport rate (ETR), (**G**,**H**) non-photochemical quenching (NPQ) of *C103* and *Gongxuan 1* under low light (LL) and high light (HL) and following transfer from low light to high light (LL−HL). Error bars show SD, *n* = 6.

The Fv/Fm varied significantly under three light treatments between the two genotypes. The shade-intolerant genotype, *C103*, showed significantly lower Fv/Fm values under LL than HL, but the shade-tolerant *Gongxuan1* showed higher Fv/Fm values under LL than HL. Two days after the transfer of the shade-adapted (LL) plant to HL conditions (LL-HL), both genotypes showed a decline in Fv/Fm values (Figure 4A,B). Compared to HL, *C103* expressed lower Fv/Fm values in LL-HL, whereas no difference was observed between HH and LL-HL in *Gongxuan1* after the 4 days of HL exposure. However, after 10 days of HL exposure, *Gongxuan1* showed higher Fv/Fm values than HL.

Both varieties showed higher PSII values under LL than HL and LL-HL on all studied days. Two days after the transfer, there was no significant difference between the PSII values under HL and LL-HL conditions. However, in the following days under LL-HL conditions, the PSII values of the two genotypes were lowest in both genotypes, showing an increased number of closed reaction centers.

We found that both varieties showed significantly higher ETR in HL than in LL throughout the experiment. After two days under LL-HL, there was a sudden increase in ETR in LL-HL compared to LL. Two varieties showed steady recovery in the following days, but neither could completely recover their HL levels. The transfer of shade-adapted LL plants to HL induced significant NPQ in both genotypes. However, the NPQ values of the shade-tolerant *Gongxuan 1* were significantly higher than those of the shade-intolerant C103.

### 3.4. Growth Characteristics

As shown in Table 1, soybean plants exposed to LL conditions showed reduced total biomass and stem diameter and an elongated stem. However, the exposure to increased light intensity under LL-HL conditions caused a steady increase in biomass, stem girth and plant height, demonstrating the recovery growth of both genotypes. Compared to *C103* under LL (at 0 days) and LL-HL (at 10 days), *Gongxuan 1* always showed shorter plant height and higher biomass and stem diameter, indicating its higher tolerance to shade stress.

### 3.5. Leaf Morphological Traits

The plants of both genotypes under LL conditions showed significantly higher SLA and LAR and lower LA in LL compared to HL (Table 2). After 10 days of transference, the SLA and LAR of LL-HL were still much higher than in HL, but there was no significant difference in the LAs of the plants between HL and LL-HL. *Gongxuan 1* showed higher LA than *C103* in LL at 0 days and in LL-LH at 10 days after exposure to high light.

## 4. Discussion

### 4.1. Physiological Acclimation

Plants adapt to shade through either shade tolerance [14] or shade avoidance mechanisms [19,20]. Shade tolerance mechanisms, which can help plants survive under low-light conditions, and to increase light harvesting or light use efficiency. These mechanisms include increasing chlorophyll (Chl) content, increasing specific leaf area and reducing the Chl a/b ratio [21]. For instance, shaded leaves show higher Chl content per leaf mass and a lower Chl a/b ratio owing to the bigger chloroplast size and lower number of chloroplasts per unit area [22,23]. Similarly, our results showed that both soybean varieties showed shade tolerance features by adjusting the Chl content and chlorophyll a/b ratio after transferring to HL. However, *Gongxuan1* showed more rapid recovery than *C103* as it is more effective in translocating the nitrogen from light-harvesting components (such as chlorophyll and carotenoids) to carboxylation components (such as RuBP carboxylation and regeneration enzymes) under changing light conditions. These results reflect that soybean can adjust plant chlorophyll content dynamically to adapt to different light conditions.

Light intensity is one of the most important factors affecting the photosynthetic efficiency of plants. Below the light saturation point, any decrease in light intensity decreases leaf *P_n_* [24], while exposure to higher light intensity may also decrease *P_n_* through photoinhibition. Consistent with the previous studies, the shade conditions in the present study reduced the photosynthesis rate and stomatal conductance of both the genotypes (Figure 2A–D), albeit more obviously in *C103* [10,25]. In maize–soybean relay strip intercropping, shade-grown soybean seedlings can be exposed to full sunlight after maize harvest, which may induce photoinhibition and cause a decrease in *P_n_*, particularly in the genotypes with less acclimation capacity [26,27]. Similarly, we also observed a post-transfer decrease in *P_n_* in both soybean varieties, possibly due to photoinhibition induced by the sudden exposure to increased light intensity. The shade-intolerant variety, *C103*, showed a greater decline in *P_n_* than the shade-tolerant *Gongxuan1,* which indicates its sensitivity to changing light conditions. *Gongxuan1*, selected from the local natural population in the 1990s, expressed lower *P_n_* than *C103* under high light and light transfer, but it recovered to the control level, which is consistent with its lower levels of photoinhibition. In contrast, in recent years, the development of C103, as an artificial breeding variety, has focused more on improving its photosynthetic capacity than on acclimation to light transfer. Previous research suggested that increased *P_n_* under high light acclimation could be due to the accumulation of photosynthetic enzymes in mature leaves [28] or the rearrangement of chloroplasts in palisade cells to cover a larger cell membrane area [29]. Therefore, the increased *P_n_* after the transfer of shade-adapted soybean plants to higher light intensity might be linked to the increased activity of ribulose-1,5-bisphosphate carboxylase/oxygenase in the chloroplasts, the number and volume of chloroplasts or their combined impact. Since photosynthesis is crucial for yield formation, the soybean genotypes with the ability to overcome photoinhibition and acclimatize to light conditions after maize harvest show higher recovery growth by gradually increasing their *P_n_* under the increased light intensity.

Chlorophyll fluorescence parameters can explain the adaptability of plants to light intensity changes from the angle of internal changes caused by photosynthesis [30]. Some studies have found that the sudden exposure of shade-grown seedlings to full sunlight resulted in an immediate and substantial reduction in Fv/Fm and PSII, followed by a gradual recovery [31,32,33]. In this study, we also noticed a decline in Fv/Fm and PSII after 2 days of exposure to high light intensity. This illustrated that the photochemical efficiency of the shade-adapted soybean was inhibited when it was exposed to natural light. This decrease in Fv/Fm in *C103* was larger than that in *Gongxuan1*, indicating that *C103* suffered more severe photoinhibition. Moreover, the higher Fv/Fm of *Gongxuan1* under low light compared to high light is consistent with its better adaptability to shade [13].

NPQ is an important pathway to the dissipation of energy [34]. Combined with previous studies, we found that shade reduces the NPQ of the two soybean varieties, which shows that energy dissipation is relatively low in soybeans under shade [35]. The increase in NPQ in the two soybean varieties after transference (Figure 4) indicated that energy dissipation is important for the recovery of Fv/Fm, especially for the shade-tolerant *Gongxuan1*, which showed higher NPQ in LL-HL than *C103*, indicating that *Gongxuan1* may have greater capability for energy dissipation after transfer. This article mainly analyses the photosynthetic electron transport chain to explain the cause of the differences in photosynthetic capacity between two soybean varieties after light transfer, not involving yield and its components; the influence on yield under different light conditions still needs further research.

### 4.2. Morphological Acclimation

The shade avoidance mechanism mainly shows morphological plasticity, and refers to the ability of plants to alter their morphology in response to light changes in their environment [36]; it can help plants escape from shade and likely increase light capture, inducing responses such as enhanced stem and petiole elongation, higher dry mass allocation to the stem than to the leaf and root, and the development of small leaf angles [19,20].

Consistent with the previous studies, stem elongation and reduced stem diameter were observed in soybean plants to enable them to reach for the light under shade. Moreover, our results showed that shade caused a reduction in plant biomass and leaf area and increased SLA and LAR (Table 1) [8,14,37]. The leaf area is also an important factor affecting plant light interception and biomass [38]. Compared to the intolerant variety, *C103*, the shade-tolerant *Gongxuan1* showed larger LA in the shade, indicating that *Gongxuan1* can intercept more light, enabling it to adapt to shade conditions. This is consistent with its shorter height under shade conditions. Taken together, these results show that better light interception mediated by higher leaf area in *Gongxuan1* helped it avert shade avoidance syndrome and accumulate more biomass than shade-susceptible *C103*. After light transfer, the two soybean genotypes showed recovery growth, especially for biomass and leaf area accumulation (Table 1). Studies have found biomass partition to the leaf and leaf area after recovery from reduced light conditions in intercropping systems [13,39,40]. Similarly, the recovery growth observed in the present study was mainly due to the rapid increment in leaf area and the maintenance of carbon gain balance. Although the increase in plant height under LL-HL was slower than in LL (Table 1), plant height and stem diameter still expressed irreversible differences between LL and HL, even after transfer. Altogether, the larger biomass and leaf area in *Gongxuan1* than in *C103* indicate that it has a strong recovery capacity and is more suitable for intercropping. The results of this study confirm the difference in the adaptability of the photosynthetic characteristics of two soybean varieties with contrasting shade tolerance when transferred from low light to high light, and provide a reference for improving the yield of intercropped soybean by screening or breeding more suitable varieties.

## 5. Conclusions

This study observed the genetic variation within soybean in acclimation to light transfer. The results showed that the photosynthetic capacity (*P_n_*, Fv/Fm and PSII) of *Gongxuan1* can recover to the control level after transfer to increased light intensity conditions. Moreover, *Gongxuan1* showed 46.1% higher NPQ, 24.1% more biomass and 10.9% more leaf area than *C103*. Therefore, it can be concluded that the acclimation capacity of the shade-intolerant variety, *C103*, is lower than that of the shade-tolerant variety *Gongxuan1*. Thus, *Gongxuan1* appears to be more suitable for the changing light environment of relay intercropping. This provides a new target for soybean screening or breeding for intercropping in the future.

## Figures and Tables

**Table 1:** Biomass, plant height and stem diameter of soybean grown under high light (HL) and low light (LL) and following transfer from low light to high light (LL-HL).

			Biomass	Plant Height	Stem Diameter
Time	Variety	Treatment	(g plant^–1^)	(cm)	(mm)
0 day					
	*Gongxuan1*	HL	10.4 ± 0.6 a	35.0 ± 2.7 c	6.9 ± 0.4 a
		LL	4.7 ± 0.8 b	57.5 ± 4.9 b	3.5 ± 0.1 c
	*C103*	HL	9.8 ± 0.5 a	52.5 ± 4.0 b	5.5 ± 0.2 b
		LL	3.2 ± 0.4 c	96.5 ± 4.4 a	2.9 ± 0.2 d
	ANOVA (*F*-value)				
	Treatment (T)		56.93 **	12.31 **	16.42 **
	Variety (V)		0.14	0.15 *	0.05
	T × V		0.13 **	0.04 **	0.03 *
10 days					
	*Gongxuan1*	HL	13.7 ± 1.0 a	45.0 ± 4.8 d	7.1 ± 0.5 a
		LL	7.2 ± 0.6 e	78.0 ± 6.8 b	4.8 ± 0.4 c
		LL-HL	11.3 ± 0.4 c	69.0 ± 4.6 c	5.2 ± 0.5 b
	*C103*	HL	13.0 ± 1.1 b	65.0 ± 6.4 c	6.2 ± 0.2 a
		LL	6.8 ± 0.3 e	105.0 ± 5.6 a	4.0 ± 0.3 d
		LL-HL	9.1 ± 1.1 d	102.3 ± 4.9 b	4.3 ± 0.1 c
	ANOVA (*F*-value)				
	Treatment (T)		43.12 **	10.42 **	12.32 *
	Variety (V)		1.85 *	1.95 **	0.61 *
	T × V		1.68 *	0.56 *	0.40

* and **: significant at *p* ≤ 0.05 and *p* ≤ 0.01, respectively. Data represent mean ± SD of three replicate plots (*n* = 6). Values followed by different letters in the same column are significantly different at the 0.05 probability level in *ANOVA*.

**Table 2:** Leaf area ratio (LAR), specific leaf area (SLA) and total leaf area (LA) of soybean plants grown under high light (HL) and low light (LL) and following transfer from low light to high light (LL-HL).

			LAR	SLA	LA
Time	Variety	Treatment	(cm^2^ g^–1^)	(m^2^ kg^–1^)	per Plant (cm^2^)
0 day					
	*Gongxuan1*	HL	157.5 ± 4.2 b	26.6 ± 2.0 b	1615.8 ± 108.2 a
		LL	202.8 ± 6.5 a	34.0 ± 1.0 a	861.3 ± 197.9 b
	*C103*	HL	149.2 ± 5.3 b	27.2 ± 0.6 b	1470.1 ± 167.0 a
		LL	184.1 ± 3.0 a	41.0 ± 2.6 a	571.6 ± 185.0 b
	ANOVA (*F*-value)				
	Treatment (T)		32.42 *	2.41 *	4.93 *
	Variety (V)		1.70	0.73	3.39
	T × V		1.47	0.32	1.23
10 days					
	*Gongxuan1*	HL	135.2 ± 3.1 c	22.7 ± 0.6 c	1705.0 ± 123.9 a
		LL	198.3 ± 6.0 a	35.0 ± 0.8 a	1544.4 ± 127.7 b
		LL-HL	148.8 ± 8.9 b	30.5 ± 1.6 b	1664.8 ± 111.2 a
	*C103*	HL	126.0 ± 1.8 c	20.8 ± 0.6 c	1632.5 ± 114.9 a
		LL	203.5 ± 5.6 a	35.5 ± 1.5 a	1386.4 ± 91.1 c
		LL-HL	163.2 ± 9.5 b	32.9 ± 1.0 b	1502.0 ± 100.6 b
	ANOVA (*F*-value)				
	Treatment (T)		32.42 **	1.10 **	4.93 *
	Variety (V)		0.13	0.04	0.18
	T × V		0.11	0.02	0.06

* and **: significant at *p* ≤ 0.05 and *p ≤* 0.01, respectively. Data represent mean ± SD of three replicate plots (*n* = 6). Values followed by different letters in the same column are significantly different at the 0.05 probability level in *ANOVA*.

## Data Availability

The data that support this study may be shared upon reasonable request to the corresponding author, if appropriate.
